# Associations between Paternal Anxiety and Infant Weight Gain

**DOI:** 10.3390/children8110977

**Published:** 2021-10-28

**Authors:** Nobutoshi Nawa, Angela C. B. Trude, Maureen M. Black, Lorenzo Richiardi, Pamela J. Surkan

**Affiliations:** 1 Department of Global Health Promotion, Tokyo Medical and Dental University, Tokyo 113-8519, Japan; 2 Department of Medical Education Research and Development, Tokyo Medical and Dental University, Tokyo 113-8519, Japan; 3 Department of Pediatrics, School of Medicine, University of Maryland, Baltimore, MD 21201, USA; angela.trude@nyu.edu (A.C.B.T.); mblack@som.umaryland.edu (M.M.B.); 4 RTI International, Research Triangle Park, NC 27709, USA; 5 Cancer Epidemiology Unit, Department of Medical Sciences, CPO-Piemonte, University of Turin, 10126 Turin, Italy; lorenzo.richiardi@unito.it; 6 Department of International Health, Bloomberg School of Public Health, Johns Hopkins University, Baltimore, MD 21205, USA; psurkan@jhu.edu

**Keywords:** anxiety, pediatric obesity, longitudinal studies

## Abstract

The aim of this study was to examine the relationship between parental anxiety (father-only, mother-only, or both) and infant weight change. We performed a secondary data analysis among 551 children in the Avon Longitudinal Study of Parents and Children, a birth cohort with weight measurements collected prospectively at 4, 8, and 12 months of age. Paternal and maternal anxiety symptoms were based on the eight-item anxiety subscale of the Crown-Crisp Experiential Index. Scores in the top 15% at 8 weeks postpartum were classified as high anxiety. Generalized Estimating Equations were employed to estimate the joint association between parental anxiety and change in child weight-for-age z-score. Children who had fathers, but not mothers, with anxiety showed a 0.15 (95% CI: 0.01, 0.29) greater increase in weight-for-age z-score than children with neither parent anxious. This result suggests that paternal anxiety, not maternal anxiety, was associated with increases in child weight gain in the first year of life. Public health practitioners and clinicians should consider the use of robust measures of both maternal and paternal anxiety in the postpartum period, in addition to the suggested screening for postpartum depression. Given the limitations of the study, this study should be considered preliminary and hypothesis generating.

## 1. Introduction

Rapid weight gain in the first year of life can lead to adverse health consequences, including increased risk for obesity and other chronic diseases [1,2,3]. Prior studies have linked maternal depression and anxiety to excess infant weight gain [4,5]. Plausible explanations for this association include changes in maternal feeding practices (e.g., restrictive feeding practices, nonresponsive feeding styles, reduced breast-feeding duration) that are known to be associated with excess infant weight gain [6,7,8,9,10,11]. Although many studies have examined the relation between maternal mental health and infant weight gain [4,5], paternal anxiety has been understudied. Paternal anxiety is estimated to occur in 4.0–16.0% of fathers prenatally and between 2.5% and 18.0% postnatally in several high-income countries [12], suggesting that paternal anxiety may also play an important role in excess infant weight gain.

From a family perspective, anxiety in either parent may influence the other parent’s relationship with the child [13]. For example, fathers with anxiety may influence maternal feeding practices associated with excess infant weight gain. Paternal and maternal anxiety are positively correlated with child anxiety [14,15], which in turn may influence children’s self-regulation and fussiness during mealtimes [16]. Thus, anxiety in both mothers and fathers could be associated with changes in children’s weight over time. Parental anxiety may be triggered by multiple factors, including concerns about abnormal infant weight gain, or feeding problems [17]. Since feeding difficulties, which can be caused by, for example, fussiness in infants, may induce anxiety in parents [17], we considered it possible that parental anxiety related to feeding difficulties could be associated with changes in feeding behaviors (e.g., parents changing the frequency and method of feeding) and in turn, related to infant weight gain. However, as we have shown previously, maternal anxiety, regardless of perceived feeding difficulties may also lead to infant weight gain [5]. As the mechanism of the association between parental anxiety and excess infant weight gain may differ depending on the presence or absence of feeding difficulties, we examined whether maternal-reported feeding difficulties modified the association between parental anxiety and excess infant weight gain. Longitudinal designs that assess relations between parental anxiety evaluated prior to infant weight measurements, infant weight gain, and feeding problems are particularly relevant, but rare.

In this study, we analyzed the Avon Longitudinal Study of Parents and Children (ALSPAC) cohort data [18] collected at multiple time points during the first year of life. In the ALSPAC cohort, fathers and mothers reported symptoms of general anxiety using the eight-item subscale of generalized anxiety from the Crown-Crisp Experiential Index prior to the measurement of infant weight gain [19,20,21,22,23,24,25,26,27,28]. High anxiety symptoms were defined as the top 15% (85th percentile or higher) of parents’ Crown-Crisp Experimental Index scores at 8 weeks postpartum, and low anxiety symptoms were defined as those below the 85th percentile at 8 weeks postpartum. This cutoff value was based on the prevalence of maternal anxiety before and after childbirth in the UK [29] and has been used previously in studies of maternal and paternal anxiety using ALSPAC data [21,22,23,24,25,26,30].

The objective of this study was to investigate if parental anxiety (father-only, mother-only, or both) was related to weight change in infancy (defined by change in infant weight-for-age z-scores). A secondary aim was to examine if mother-reported feeding difficulties modified the association between parental anxiety and excess infant weight gain. Given the association between child’s sex [31] and the mother’s postpartum depression, as well as differences in the child growth depending on child’s sex, we also examined whether child’s sex modified the association between parental anxiety and excess infant weight gain.

## 2. Materials and Methods

### Sample

We analyzed data from participants in the Children in Focus (CiF) sub-study, which corresponds to 10% of the ALSPAC cohort, who attended clinics at the University of Bristol. Initially, ALSPAC recruited 14,541 pregnant women in Avon who were expecting to deliver between 1 April 1991 and 31 December 1992. A total of 14,541 pregnant women who were enrolled in the ALSPAC study returned at least one questionnaire or attended a “Children in Focus” clinic by 19 July 1999. Of these initial pregnancies, there were 14,676 fetuses, resulting in 14,062 livebirths and 13,988 children who were alive at 1 year of age [32,33] (the study website contains details of all available data through a fully searchable data dictionary and variable search tool [34]). We excluded infants with major congenital abnormalities, cerebral palsy, and non-singleton births [35]. The CiF group was chosen at random from the final 6 months of ALSPAC births, corresponding to 1432 families who attended at least one clinic visit. We excluded mothers who had moved out of the area, who were lost to follow-up, or who were enrolled in another study of infant development conducted in Avon [18,36,37]. Our analysis focused on infant weight change between the median ages of four and 12 months of age. We chose this age group because rapid weight gain during infancy is associated with later child obesity and other chronic diseases [1,2,3]. While CiF data were collected at various time intervals between 4 and 61 months of age, we included infants who were weighed during the earlier assessments conducted at the median ages of 4, 8, and 12 months, resulting in an analytical sample of 551 infants. The ALSPAC Ethics and Law Committee and the Local Research Ethics Committees approved this study.

## 3. Measures

### 3.1. Independent Variable

#### Parental Anxiety Symptoms

Given that anxiety among mothers and fathers during the perinatal period is common in high-income countries [12,29,38,39], in this study we focused on parental anxiety symptoms. Maternal and paternal anxiety were based on an eight-item subscale of general anxiety from the Crown-Crisp Experiential Index [19,20,21,22,23,24,25,26,27,28], reported by mothers and fathers. Examples of items in the subscale include “Do you feel upset for no obvious reason?” and “Do you feel strung-up inside?” Scores range from 0 to 16 with higher scores indicating more symptoms of anxiety. Parents who scored in the top 15% (85th percentile or higher) on the Crown-Crisp Experimental Index were considered to have high symptoms of anxiety (referred to as having ‘high anxiety’). Parents with scores below the 85th percentile were considered to have low symptoms of anxiety (referred to as having “low anxiety”). This cutoff was derived from the prevalence of anxiety among antenatal and postnatal mothers in the UK [29], and has been previously used in research on maternal and paternal anxiety using the ALSPAC data [21,22,23,24,25,26,30]. Our prior study of the relationship between maternal anxiety and gain in child BMI also used this cutoff [5]. We used data at 8 weeks postpartum for parental anxiety. The 85th percentile value of the Crown-Crisp Experimental Index at 8 weeks postpartum was 5 and 6 points for fathers and mothers, respectively. Maternal and paternal anxiety were dichotomized and analyzed as categorical variables with four categories, i.e., by creating combinations of paternal and maternal anxiety (high anxiety in mothers only, high anxiety in fathers only, high anxiety in both parents, and low anxiety in both parents) for ease of interpretation.

### 3.2. Moderator

#### Feeding Difficulties at the 6-Month Assessment

Feeding difficulties were assessed by asking mothers: “Do you feel you have ever had difficulties feeding your baby?” at the 6-month assessment. Response categories include “no difficulty”, “some difficulty”, and “yes, great difficulty”. This scale has been used in previous ALSPAC studies [40,41]. We dichotomized this variable based on previous literature [40,41] and defined a priori: “no difficulty” (coded as 0), and “with difficulty” (coded as 1), which combined some difficulty and great difficulty responses. The prevalence of feeding difficulties at 6 months was 32.3%.

### 3.3. Covariates

Covariates were maternal age, pre-pregnancy self-reported weight and height, education level, parity and race/ethnicity, paternal age, education level, and marital status (all assessed during pregnancy), and child birthweight, child gestational age, and sex (all assessed at birth). Feeding method (bottle feeding, breastfeeding, both bottle feeding and breastfeeding, and other) was assessed at 4 weeks postpartum. Variables were chosen based on associations reported previously [5,42]. Maternal age, pre-pregnancy self-reported weight and height, parity, paternal age, child birthweight, and child gestational age were used as continuous variables, while maternal education level (lower than O-level, O-level, A-level, degree), race/ethnicity (other race/ethnicity, White), paternal education level (lower than O-level, O-level, A-level, degree), marital status (‘single’ included never married, widowed divorced and separated; ‘married’ included both first or later marriages), and child sex (male, female) were treated as categorical variables. In terms of parental education level, A level or ‘advanced level’ is the level of a post-compulsory education usually taken at age 18. O level or ‘ordinary level’ is at the end of compulsory education usually taken at age 16 [43,44]. We did not have information on whether mothers or fathers were living with their children during the study period. It is also possible that some of the partners who responded to the partner questionnaire from which we extracted information on paternal anxiety were not the biological fathers of the children. Therefore, we were not able to take these factors into account in the analysis.

### 3.4. Dependent Variable

#### Child Weight

Child weight was measured in clinics at the University of Bristol to the nearest 0.1 kg when children (weighed wearing only underwear) were median ages of 4 (min: 3.5, max: 4.8), 8 (min: 7.4, max: 9.5), and 12 (min: 11.8, max: 13.9) months. Measurements were taken using a Fereday 100 kg combined scale at 4 months, a Soenhle scale or a Seca 724 scale at 8 months, and a Seca 724 or a Seca 835 Scale at 12 months. Considering that the data were collected in the UK in the 1990s, we used the UK 1990 growth reference curves [45,46] to convert data to weight-for-age z-scores.

## 4. Statistical Analyses

All statistical analyses were conducted using R, version 3.4.3. We used Generalized Estimating Equations (GEE) with the “gee” package, version 4.13–19 to estimate associations between parental anxiety and child change in weight-for-age z-score from the first time point (median age of 4 months) to the third time point (median age of 12 months) by accounting for within-individual associations between repeated measures from the same child [47]. We included an interaction term between parental anxiety and child age in the model to assess whether child weight-for-age z-score trajectories varied by parental anxiety. We centered child age at 4 months and scaled so that a one-unit change in age could be interpreted as a change over 4 months. We also explored heterogeneity by child sex. Since results did not differ by sex, child sex was included as a covariate.

We investigated if the association between parental anxiety (at 8 weeks postpartum) and infant weight gain from 8 to 12 months was modified by feeding difficulties when children were 6 months old using GEE analyses. We included an interaction term between combinations of paternal and maternal anxiety (coded as maternal-only high anxiety, paternal-only high anxiety, high anxiety in both parents, low anxiety in both parents (reference group)) and feeding difficulties in the model. Age was also included in a three-way interaction term to assess whether the rate of change in child weight-for-age z-scores varied by child parental anxiety status with or without child feeding difficulties.

## 5. Missing Data

Missing values ranged from 0.4% for birthweight to 29.9% for paternal age. To handle missing values on covariates, we used multiple imputation and created 50 imputed datasets using the “mice” package, version 2.46.0 [48]. The results of analyses using all the imputed datasets were combined using Rubin’s rules for multiple imputation [49].

## 6. Results

As shown in Table 1, mean ages of mothers and fathers were 28.5 years (SD: 4.5) and 31.5 years (SD: 5.5), respectively. Most mothers were White, and 78.9% of mothers were married. The prevalence of high anxiety was 21.2% among mothers and 14.2% among fathers at 8 weeks. In total, 64.9% of families reported low anxiety in both parents, while 7.2% of families reported high anxiety in both parents. One third of the mothers reported feeding difficulties when the child was 6 months old.

Table S1 shows raw data (without fitting a regression model) of the mean child weight-for-age z-score measured at the median ages of 4, 8, and 12 months by parental anxiety in the unimputed dataset. The weight-for-age z-score of children who had mothers (but not fathers) with high anxiety increased from −0.59 at the age of 4 months to −0.23 at the age of 12 months, whereas the weight-for-age z-score of children of fathers (but not mothers) with high anxiety increased from −0.13 to 0.23 over this same time period.

Table 2 shows the regression model-derived associations of paternal and maternal anxiety with change in child weight-for-age z-score from the first time point (median age 4 months) to the third time point (median age of 12 months). In the imputed samples, 7.2% of children had both parents with high anxiety. After covariate adjustment, children with parents for whom only fathers had high anxiety (i.e., the mothers did not have high anxiety) showed greater increases in weight-for-age z-score for each 4-month increase in child age (β = 0.15, 95% CI: 0.01, 0.29, i.e., the coefficient for the child age* high anxiety interaction in fathers only) compared to children for whom both parents were in the low anxiety group.

Table 3 shows the associations of paternal and maternal anxiety with change in child weight-for-age z-score from the second time point (median age 8 months) to the third time point (median age 12 months), stratified by feeding difficulties when children were 6 months old. Among children with feeding difficulties, high paternal anxiety was associated with a 0.24 (95% CI: 0.02, 0.46) greater increase in weight-for-age z-score compared to children whose fathers and mothers had low anxiety. The *p*-value for the three-way interaction term (child feeding difficulties by paternal anxiety by child age) was 0.36.

## 7. Discussion

In this study, we found that children who had fathers but not mothers with high anxiety showed a clinically significant increase in weight-for-age z-score over 8 months, compared to children with neither fathers nor mothers with high anxiety. This pattern was replicated among children with feeding difficulties, where high paternal anxiety was associated with a clinically significant increase in weight-for-age z-score compared to children whose fathers and mothers had low anxiety.

Maternal and paternal anxiety during the perinatal period is common in high-income countries [12,29,38,39]. A prior study found that pre- and post-natal maternal anxiety was associated with an increase in child BMI at age 2 years (25–31 months of age), with no consideration of paternal anxiety [5]. To date, we are not aware of any studies that have assessed the relationship between paternal anxiety and infant weight gain. Given the relative paucity of existing data on the influence of paternal (relative to maternal) anxiety, these findings add to the literature by highlighting the potential importance of paternal anxiety on infant development.

A possible explanation for the finding that high anxiety in fathers but not in mothers was associated with increases in child weight-for-age z-score may be differences in the sex-specific manifestations of anxiety [50]. For example, qualitative studies on fathers have reported difficulties in balancing work and family life (especially sleeping and feeding patterns) in the presence of psychosocial distress [51,52]. In a qualitative study in the UK, fathers attributed higher levels of anxiety to greater financial pressure after becoming parents [52], thus potentially influencing household food security and quality of available food that could lead to child weight gain. We lacked information on unemployment and food security and could not study this issue further. Furthermore, the underlying causes of anxiety may be different for mothers and fathers. We did not find a joint effect of maternal and paternal anxiety on infant weight gain trajectory, possibly due to the low prevalence of households with high anxiety among both mothers and fathers (7.1%). It is possible that paternal mental health symptoms during the perinatal period may pose negative consequences for the whole family including family relationships as well as the health of their spouses and children [53].

Paternal mental health problems can lead to marital conflicts that can negatively impact the entire family [54]. In most countries and cultures, fathers are becoming more involved in child rearing than previously [54]. Given that paternal psychological wellbeing may influence fathers’ parenting behaviors and the family as a whole, paternal wellbeing may play a significant role on children’s weight, growth, and development.

We also assessed whether infant feeding difficulties modified the association between high paternal anxiety and infant weight gain, given the association between paternal anxiety and children’s self-regulation and fussiness during mealtimes [14,15,16]. Our results suggest that mother-reported feeding difficulties did not modify the association. This is perhaps partially explained by the use of a single-item feeding difficulties measure. In addition, feeding problems are relatively common at 6 months, when infants transition from a liquid diet of breast milk or formula via breast or bottle to a complementary diet of semi-solid foods that are often spoon-fed or self-fed [55]. Parents may have anticipated a transition and had strategies to deal with the feeding problems at 6 months [55]. Future studies should use robust and validated methods to evaluate feeding difficulties, from the perspectives of both parents.

Our findings have clinical implications. In this study, we found that children who had fathers but not mothers with high anxiety showed a clinically significant increase in weight-for-age z-score over 8 months, compared to children with neither fathers nor mothers with high anxiety. If this increase in weight gain continues over 2 years, it would translate to a weight gain of about 0.90 SD, exceeding the criterion for rapid weight gain of 0.67 SD over 2 years [56,57,58]. Guidelines from the National Institute for Health and Care Excellence (NICE) in the UK recommends screening for maternal postpartum depression and anxiety and calls attention to the importance of considering the mental health needs of fathers or partners [29]. The American Academy of Pediatrics recently recommended routine screening for maternal postpartum depression at well-child visits at 1, 2, 4, and 6 months of age [59]. Their statement also recommends screening the partner for depression at the 6-month visit using the Edinburgh Postpartum Depression Scale (EPDS) [59,60]. Screening and referrals for parental mental health may protect children from the negative consequences of exposure to mental illness in the home and associated adverse current and later health consequences [59,61]. However, the EPDS was not designed to measure anxiety and includes only three anxiety-specific items [60]. Given the association that we observed between high paternal anxiety and infant weight gain as well as other research on paternal anxiety and child development [15,54,62], clinicians should consider screening both mothers and fathers for anxiety in the postpartum period (for possible intervention if identified) [63,64], in addition to screening for depression at 6-months postpartum. Interventions may include digital mental health using web-based information and communication technologies that have been reported to be effective in a recent systematic review and meta-analysis [63].

Study strengths include father-reported information on fathers’ emotional state, which has been largely neglected in the literature, adding to the value of this investigation. Another major advantage is the use of the relatively large longitudinal ALSPAC dataset. As parental anxiety could be triggered by concerns about abnormal weight and/or growth of the child, a longitudinal design is needed to assess the relation between parental anxiety evaluated prior to infant weight measurements and infant weight gain. By analyzing data from the longitudinal ALSPAC dataset, we found that paternal anxiety was associated with increases in infant weight gain in the first year of life, to a degree that exceeded the definition of rapid weight gain.

Our study has several limitations. First, we did not have information on whether mothers and fathers were still married or co-habitating over the course of data collection (from child age 4 to 12 months). It is also possible that not all the participants who responded to the partner questionnaire (from which we extracted information on paternal anxiety) were biological fathers of the child. Second, anxiety was based on a brief symptom checklist, rather than a diagnostic interview. Third, the choice of 85th percentile cutoff for paternal anxiety was based on use of this cutoff to define anxiety in prior research [28], including an ALSPAC study of paternal anxiety [30]. In the absence of established cutoffs for paternal anxiety, future validation studies should establish appropriate cutoffs to be used. Fourth, we did not have information on the content of the milk that was being bottle fed (breast milk vs. formula). Fifth, in order to calculate the error in the measurement of infant body weight, it is necessary to take multiple measurements, but since no such data were collected, measurement error cannot be calculated and is unknown. Sixth, the mechanism of the association between paternal anxiety and infant weight gain is not yet known. Future research could examine whether fathers with anxiety increase their involvement with bottle feeding due to concern over poor weight gain, thereby increasing the risk for excess infant weight gain. Future research should also consider children’s behavioral and health factors that may influence feeding. Finally, ALSPAC is a valuable longitudinal data set that measures anxiety in both fathers and mothers as well as child weight over time using objective measures. However, the cohort represents a predominantly homogeneous population of White parents in Britain in the 1990s. Compared to the 1990s, society has changed, including changes in family dynamics (e.g., increases in fathers’ involvement with infant care, greater proportions of fathers who take paternity leave during the infant’s first year) and changes in the sources of parental anxiety (e.g., social media, smart phones). In addition, the stratified analysis by feeding difficulties used a three-way interaction term, which has low statistical power. Therefore, this study should be considered hypothesis-generating and call attention to the potential role of paternal anxiety in current times. Future studies with larger sample sizes using WHO standards to convert data to weight-for-age z-scores and more recent samples are needed to confirm if these findings are generalizable to other and contemporary populations. Studies are also warranted to determine if paternal anxiety in the early postpartum period continues to be associated with increased weight gain over time. Given these limitations, the results of this study should be considered preliminary.

## 8. Conclusions

In conclusion, we found that infants who had fathers but not mothers with high anxiety showed greater increases in weight-for-age z-score than infants with both parents reporting low anxiety. Additional research is needed to examine the mechanisms driving the association. Given the positive association between paternal anxiety and children’s weight gain trajectory, future research and clinical work should assess fathers’ psychological wellbeing and longitudinal impact on children’s health and development.

## Figures and Tables

**Table 1:** Characteristics of study participants (total *n* = 551).

Variable	*n*	%
Parental characteristics		
Maternal education ^a^		
Lower than O-level	105	19.1
O-level	198	35.9
A-level	150	27.2
Degree	62	11.3
Missing	36	6.5
Paternal education ^a^		
Lower than O-level	91	16.5
O-level	105	19.1
A-level	151	27.4
Degree	89	16.2
Missing	115	20.9
Maternal race/ethnicity		
Other than White	8	1.5
White	527	95.6
Missing	16	2.9
High maternal anxiety at 8 weeks postpartum		
Yes	117	21.2
No	411	74.6
Missing	23	4.2
High paternal anxiety at 8 weeks postpartum		
Yes	78	14.2
No	339	61.5
Missing	134	24.3
Marital status		
Single	105	19.1
Married	435	78.9
Missing	11	2.0
Feeding method at 4 weeks postpartum		
Breastfeeding	257	46.6
Bottle feeding	205	37.2
Breastfeeding and bottle feeding	71	12.9
Other	4	0.7
Missing	14	2.5
Feeding difficulties at the age of 6 months		
Yes	178	32.3
No	350	63.5
Missing	23	4.2
	Mean	SD
Maternal age (years)	28.5	4.5
Paternal age (years)	31.5	5.5
Maternal pre-pregnancy weight (kg)	63.2	11.4
Maternal height (cm)	164.4	6.2
Maternal pre-pregnancy body mass index (kg/m^2^)	23.5	4.2
Parity	0.8	1.0
Child characteristics		
	*n*	%
Sex		
Male	284	51.5
Female	267	48.5
Missing	0	0
Low birthweight		
Yes	17	3.1
No	532	96.6
Missing	2	0.4
Preterm birth		
Yes	18	3.3
No	533	96.7
Missing	0	0
	Mean	SD
Birthweight (g)	3437	491
Gestational age (weeks)	39.5	1.5
Child weight-for-age z-score at 4 months ^b^	−0.19	0.92
Child weight-for-age z-score at 8 months ^b^	0.00	1.00
Child weight-for-age z-score at 12 months ^b^	0.09	0.98
Child weight-for-age z-score change from 4 to 8 months ^a^	0.19	0.61
Child weight-for-age z-score change from 8 to 12 months ^a^	0.09	0.46

SD = standard deviation ^a^ A level or ‘advanced level’ is the level of a post-compulsory education usually taken at age 18. O level or ‘ordinary level’ is at the end of compulsory education usually taken at age 16 [43,44]. **^b^** Due to the variability in when children attended study visits, these dates represent the median value of months when the children in the sample were weighed.

**Table 2:** Categorical profiles of paternal and maternal anxiety with change in child weight-for-age z-score from the first time point (median age of 4 months) to the third time point (median age of 12 months) in the imputed dataset using GEE ^a^.

		Crude	Adjusted ^b^
	%	β (95%CI)	β (95%CI)
Child age		0.07 (0.03, 0.11) ^†^	0.05 (0.006, 0.09) ^†^
Parental anxiety			
Low anxiety in fathers and mothers	64.9%	0.00 (Reference)	0.00 (Reference)
High anxiety in fathers only	12.8%	−0.06 (−0.32, 0.21)	−0.11 (−0.36, 0.15)
High anxiety in mothers only	15.1%	−0.32 (−0.57, −0.07) ^†^	−0.28 (−0.52, −0.04) ^†^
High anxiety in both mothers and fathers	7.2%	0.26 (−0.08, 0.60)	0.20 (−0.13, 0.53)
Child age * Low anxiety in fathers and mothers		0.00 (Reference)	0.00 (Reference)
Child age * High anxiety in fathers only		0.12 (0.01, 0.24) ^†^	0.15 (0.01, 0.29) ^†^
Child age * High anxiety in mothers only		0.07 (−0.03, 0.17)	0.08 (−0.05, 0.20)
Child age * High anxiety in both mothers and fathers		−0.004 (−0.12, 0.11)	0.004 (−0.13, 0.14)

^a^ We centered age at 4 months and scaled age so that the effect of a one-unit change in age can be interpreted as the effect of a 4-month increase in age. ^b^ Adjusted for maternal age, pre-pregnancy weight, height, education, parity, race/ethnicity, marital status, paternal education, age, child birthweight, gestational age, sex, and feeding method. Child age*parental anxiety refers to the interaction term between child age and parental anxiety. ^†^ *p* < 0.05.

**Table 3:** Categorical profiles of paternal and maternal anxiety with change in child weight-for-age z-score from the second time point (median age of 8 months) to the third time point (median age of 12 months) in the imputed dataset, stratified by feeding difficulties with GEE ^a^ (because the outcome has two timepoints).

	Crude	Adjusted ^b^
	β (95%CI)	β (95%CI)
Child weight-for-age z-score change from 8 to 12 months		
***Child feeding difficulty***		
Child age	0.05 (−0.02, 0.12)	0.05 (−0.02, 0.12)
Parental Anxiety		
Low anxiety in fathers and mothers	0.00 (Reference)	0.00 (Reference)
High anxiety in fathers only	−0.38 (−0.84, 0.08)	−0.35 (−0.90, 0.19)
High anxiety in mothers only	−0.33 (−0.74, 0.07)	−0.22 (−0.59, 0.15)
High anxiety in both mothers and fathers	0.20 (−0.46, 0.86)	0.02 (−0.52, 0.56)
Child age * Low anxiety in fathers and mothers	0.00 (Reference)	0.00 (Reference)
Child age * High anxiety in fathers only	0.24 (0.02, 0.46)^†^	0.24 (0.02, 0.46) ^†^
Child age * High anxiety in mothers only	0.08 (−0.08, 0.24)	0.08 (−0.08, 0.24)
Child age * High anxiety in both mothers and fathers	0.04 (−0.16, 0.24)	0.04 (−0.16, 0.24)
***No child feeding difficulty***		
Child age	0.05 (−0.0002, 0.11)	0.05 (0.0003, 0.11) ^†^
Parental Anxiety		
Low anxiety in fathers and mothers	0.00 (Reference)	0.00 (Reference)
High anxiety in fathers only	0.13 (−0.22, 0.48)	0.09 (−0.22, 0.41)
High anxiety in mothers only	−0.16 (−0.53, 0.21)	−0.15 (−0.50, 0.19)
High anxiety in both mothers and fathers	0.26 (−0.19, 0.71)	0.23 (−0.20, 0.66)
Child age * Low anxiety in fathers and mothers	0.00 (Reference)	0.00 (Reference)
Child age * High anxiety in fathers only	0.10 (−0.08, 0.29)	0.10 (−0.08, 0.28)
Child age * High anxiety in mothers only	0.08 (−0.11, 0.27)	0.08 (−0.11, 0.27)
Child age * High anxiety in both mothers and fathers	−0.02 (−0.20, 0.16)	−0.02 (−0.20, 0.16)

The *p*-value for the interaction term among child feeding difficulty, high anxiety in fathers and child age is 0.36. ^a^ We centered age at 4 months and scaled age so that the effect of a one-unit change in age can be interpreted as the effect of a 4 month increase in age. ^b^ Adjusted for maternal age, pre-pregnancy weight, height, education, parity, race/ethnicity, marital status, paternal education, age, child birthweight, gestational age, sex, and feeding method. Child age*parental anxiety refers to the interaction term between child age and parental anxiety. ^†^ *p* < 0.05.

## Data Availability

The data used in this study is third party data and will be available from the ALSPAC Executive Committee (alspac-exec@bristol.ac.uk) upon request.

## Supplementary Materials

The following are available online at https://www.mdpi.com/article/10.3390/children8110977/s1.
