# Supplementary material for: Associations between Paternal Anxiety and Infant Weight Gain
Source: Children (Basel). 2021 Oct 28;8(11):977. doi: 10.3390/children8110977 (PMC8618665; doi:10.3390/children8110977)
Supplement: Supplementary file 1 [file children-08-00977-s001.zip › children-1418793-supplementary.pdf]

children MDPI

Table S1. Mean child weight-for-age z-score by parental anxiety measured at median ages of 4, 8, and 12 months in the original (unimputed) dataset.

Group	Child weight-for-age z-score at 4 months	Child weight-for-age z-score at 8 months	Child weight-for-age z-score at 12 months
	**Mean (SD)**	**Mean (SD)**	**Mean (SD)**
All children	-0.19 (0.92)	0.00006 (1.00)	0.09 (0.98)
Low anxiety in fathers and mothers	-0.11 (0.89)	0.04 (0.98)	0.08 (0.95)
High anxiety in fathers only	-0.13 (0.98)	0.03 (1.03)	0.23 (0.93)
High anxiety in mothers only	-0.59 (1.00)	-0.38 (1.11)	-0.23 (1.07)
High anxiety in both mothers and fathers	0.13 (0.88)	0.23 (1.07)	0.25 (1.08)
